# TSA and BIX-01294 Induced Normal DNA and Histone Methylation and Increased Protein Expression in Porcine Somatic Cell Nuclear Transfer Embryos

**DOI:** 10.1371/journal.pone.0169092

**Published:** 2017-01-23

**Authors:** Zubing Cao, Renyun Hong, Biao Ding, Xiaoyuan Zuo, Hui Li, Jianping Ding, Yunsheng Li, Weiping Huang, Yunhai Zhang

**Affiliations:** Anhui Provincial Laboratory of Local Livestock and Poultry, Genetical Resource Conservation and Breeding, College of Animal Science and Technology, Anhui Agricultural University, Hefei, China; University of Texas at Austin Dell Medical School, UNITED STATES

## Abstract

The poor efficiency of animal cloning is mainly attributed to the defects in epigenetic reprogramming of donor cells’ chromatins during early embryonic development. Previous studies indicated that inhibition of histone deacetylases or methyltransferase, such as G9A, using Trichostatin A (TSA) or BIX-01294 significantly enhanced the developmental efficiency of porcine somatic cell nuclear transfer (SCNT) embryos. However, potential mechanisms underlying the improved early developmental competence of SCNT embryos exposed to TSA and BIX-01294 are largely unclear. Here we found that 50 nM TSA or 1.0 μM BIX-01294 treatment alone for 24 h significantly elevated the blastocyst rate (*P* < 0.05), while further improvement was not observed under combined treatment condition. Furthermore, co-treatment or TSA treatment alone significantly reduced H3K9me2 level at the 4-cell stage, which is comparable with that in *in vivo* and *in vitro* fertilized counterparts. However, only co-treatment significantly decreased the levels of 5mC and H3K9me2 in trophectoderm lineage and subsequently increased the expression of OCT4 and CDX2 associated with ICM and TE lineage differentiation. Altogether, these results demonstrate that co-treatment of TSA and BIX-01294 enhances the early developmental competence of porcine SCNT embryos via improvements in epigenetic status and protein expression.

## Introduction

Genetically modified pigs generated by genomic editing technology are widely used as animal model for human diseases in biomedical researches and also as initial genetic resources for breeding in swine production [1]. Previous studies showed that homologous recombination-mediated gene targeting in embryonic stem cells are widely used to generate the transgenic model organisms [2]. Recently, nuclease-driven genomic editing technologies including zinc-finger nucleases (ZFNs), transcription activator-like effector nucleases (TALENs) and CRISPR/Cas9 in zygotes are becoming more popular for the generation of mutant animals [3]. Therefore, these novel genomic editing technologies-mediated genetic modifications in somatic cells combined with somatic cell nuclear transfer (SCNT) technology may be the primary method for generating the transgenic pigs in future.

SCNT is thought to be a promising and powerful biotechnology that the terminally differentiated somatic cells are successfully reprogrammed into the totipotent embryo by transplanting a donor nucleus into an enucleated oocyte [4,5]. Indeed, more than 20 different cloned species have been successfully generated by SCNT until now[6]. However, the cloning efficiency has been very low for many species, especially, porcine cloning efficiency is yet less than 5% [7], which significantly limits the biomedical and agricultural application of transgenic pigs. Increasing evidence suggests that defective epigenetic reprogramming of donor cell’s nuclear chromatins [8] and abnormal gene expression profiles [9] likely contribute to the overall poor cloning efficiency.

To date, a number of strategies have been developed to improve the cloning efficiency. One of the most commonly utilized strategies involves the application of small molecule inhibitors to regulate the epigenetic modifications of cloned embryos. Histone deacetylases (HDACs) inhibitors, such as TSA [10,11], Scriptaid [12], VPA [13], m-carboxycinnamic acid bishydroxamide [14], significantly increased the early or full-term developmental efficiency of porcine SCNT embryos through improvements in histone acetylation. In addition, histone methylation might affect the reprogramming efficiency of somatic cells to pluripotent or totipotent state. Recently, H3K9me3 and H3K79me3 are identified as two negative reprogramming regulators for generating the induced pluripotent stem cells in mouse [15] and human [16]. Similarly, abnormal histone methylation profiles involving H3K9me2 and H3K9me3 are discovered during porcine early SCNT embryonic development in our previous study [17]. Based on these studies, we hypothesize that abnormal histone methylation leads to the reduction of developmental efficiency of SCNT embryos. Recent studies demonstrate that histone demethylase, either KDM4A or KDM4B overexpression-mediated reduction in H3K9me3 level dramatically improves the overall mouse cloning efficiency [18,19]. Meanwhile, G9a knockdown by RNAi or the reduction of H3K9me2 level through a small molecule inhibitor BIX-01294-mediated inhibition of G9A activity significantly promotes the cloning efficiency in mouse [20] and pig [21]. However, the molecular mechanisms underlying the enhanced cloning efficiency in pig via modulating the status of histone acetylation and methylation remain largely unknown. Therefore, in the present study we attempt to elucidate the potential mechanisms involved in the improved early development of porcine SCNT embryos exposed to TSA and BIX-01294.

## Materials and Methods

All reagents were purchased from Sigma (Sigma-Aldrich, St Louis, MO) unless otherwise stated.

### Ethics statement

All experiments involving animals were conducted in accordance with the Institutional Animal Care and Use Committee (IACUC) guidelines under current approved protocols at Anhui Agricultural University. To collect *in vivo* embryos, 10 sows were injected with excess anesthetic and then sacrificed by the licensed veterinarians, whom are not coauthors in the present study. Animals used in the study were not experimentally manipulated before they were sacrificed. The study had been fully reviewed and approved by IACUC.

### *In vivo* embryo recovery

This experiment was performed as described previously [22]. Briefly, sows (Meishan breed) mated with two sexually mature and healthy boars were euthanized at a local abattoir at specific postmating time points, and their reproductive tracts including oviducts and uteri were excised. The 4-cell and blastocysts at 48 h or 144 h were quickly flushed out with pre-warmed 0.9% NaCl solution from the oviducts or uteri. Zonae pellucidae of embryos were removed by pronase treatment (0.5% pronase in Dulbecco’s phosphate buffered saline [DPBS]) prior to fixation at room temperature. Then fixed embryos were washed several times in DPBS supplemented with 0.3% polyvinylpyrrolidone (PVP), and stored short term at 4°C in DPBS for immunofluorescence analyses.

### *In vitro* maturation (IVM) of oocyte

This experiment was performed as described previously[22]. Briefly, the ovaries were collected from prepubertal gilts (Landrace×Large White×Duroc) at the Hefei Wanrun Slaughterhouse (Anhui, China), stored in saline and transported to the laboratory at 37°C. Immediately upon arrival, ovarian follicles with 3–6 mm were aspirated using a sterile 10 mL syringe with an 18 gauge needle. The aspirated follicular fluid was slowly injected into a 38.5°C, preincubated, 15 mL centrifuge tube to settle down the cumulus-oocyte complexes (COCs) for 15 min. After the removal of the supernatant, the cell pellets were diluted with oocyte washing medium (0.01% polyvinyl alcohol in DPBS) and aspirated gently. COCs with multiple layers of intact cumulus cells and uniform ooplasm were selected for IVM. After washing three times in IVM medium, groups of 50–100 COCs were plated onto four-well cell culture plates (Nunc, Roskilde, Denmark) containing 400 μL IVM medium covered with 400 μL mineral oil per well. Subsequently, the COCs were cultured for 42–44 h at 38.5°C and 5% CO_2_ in humidified air. Then matured COCs were pipetted up and down manually for 3 min in DPBS without Ca^2+^and Mg^2+^ (Gibco, Grand Isle, NY) containing 1 mg/mL hyaluronidase to remove the surrounding cumulus cells. Finally, the matured oocytes with clear perivitelline spaces, intact cell membranes, and extruded first polar body (pb1) were selected for subsequent experiments.

### *In vitro* fertilization

*In vitro* fertilization was performed as described previously [23]. Briefly, fresh semen was washed three times with DPBS supplemented with 0.1% BSA, 75 mg/mL penicillin G, and 50 mg/mL streptomycin, and centrifugation at 100 ×*g* for 3 min. After removing the supernatant, spermatozoa pellets were resuspended with fertilization medium (mTBM) containing 2 mg/mL BSA (fraction V) and 2 mM caffeine. Groups of 25 denuded oocytes were washed three times in fertilization medium and then transferred to 100 μL fertilization medium covered with mineral oil. Approximately 50 μL of the sperm suspension was added to the fertilization droplets, generating a final sperm concentration of 1.5 to 5.0 × 10^5^ sperm/mL. The oocytes were co-incubated with sperm for 4–6 h. After fertilization, the oocytes were washed three times and cultured in 400 μL porcine zygote medium (PZM-3) in four-well dishes at 38.5°C and 5% CO_2_ in humidified air.

### Preparation of donor cells

Sows were sacrificed at the Hefei Wanrun Slaughterhouse (Anhui, China), and then fetus in 35 days old was recovered and rinsed three times with PBS. After removing head, intestine, liver, heart and limbs, the left tissueswere immersed in a small amount of fetal bovine serum (FBS) after disinfection and cut into pieces at approximately 1mm^3^. The tissue blocks were evenly smeared in a dish and cultured upside down in 37°C, 5% CO_2_ and saturated humidity. After 8 h cell culture medium (15% FBS plus 85% DMEM, 0.1mM NEAA, and 0.05 mM L-glutamine) was added and the samples were cultured as mentioned above. Cell pellets were dissociated until 90% confluence. Fetal fibroblast cells at the 4^th^-8^th^ passages were used as donor cells for nuclear transfer.

### Somatic cell nuclear transfer

This experiment was performed as described previously [10]. Briefly, MII oocytes were placed in manipulation medium (2% FBS in TCM199) supplemented with 7.5 μg / mL cytochalasin B and enucleated through the aspiration of the polar body and MII chromosomes and a small amount of the surrounding cytoplasm using a 15–20 mm beveled glass pipette. A single donor cell was injected into the perivitelline space and placed adjacent to the recipient cytoplasm. The reconstructed couplets composed of a donor cell and oocyte cytoplasm were washed three times with PZM-3. Then they were moved into fresh PZM-3 droplets and recovered for 30 min. The resulting reconstructed couplets were placed in electrofusion medium for subsequent induction of simultaneous fusion and electrical activation. The reconstructed couplets were washed and incubated for 30 min in PZM-3 medium at 38.5°C and 5% CO_2_ in humidified air and evaluated for fusion using a stereomicroscope. Then fused embryos were further incubated for 4 h in chemically assisted activation medium (PZM-3 supplemented with 10 μg / mL Cycloheximide and 10 μg / mL CB) at 38.5°C and 5% CO_2_ with saturated humidity. Finally, fused embryos were washed three times using fresh PZM-3 medium and cultured in four-well plates containing PZM-3 medium at 38.5°C and 5% CO_2_ in humidified air.

### Preparation of TSA and BIX-01294 solution

Commercially available histone deacetylase inhibitor TSA (Sigma, T1952) and methyltransferase inhibitor BIX-01294 (Sigma, B9311) was dissolved in DMSO and water, respectively. TSA and BIX-01294 were then formulated to 5 mM and 1 mM stock solutions and stored at -20°C. PZM-3 was used to dilute the TSA and BIX-01294 stock solution to obtain the desired working solution. Furthermore, 1 μL DMSO or water was added into PZM-3 as the vehicle control.

### Immunofluorescence staining

This experiment was performed as described previously [17]. Briefly, embryos were treated with 0.5% pronase solution to remove the zona pellucida. Zona-free embryos were washed three times with DPBS containing 0.3% PVP and fixed in 4% paraformaldehyde in DPBS for 15 min. The fixed embryos were permeabilized with 0.5% Triton X-100 in DPBS for 30 min at room temperature, then washed briefly with DPBS containing 0.3% PVP. Embryos were incubated in 2 N HCl solutions at room temperature for 15 min and blocked with 1% BSA in DPBS overnight at 4°C. Embryos were subsequently incubated in blocking buffer containing primary antibodies against 5mC (Abcam, ab10805, 1:200), H3K9me2 (Abcam, ab1220, 1:200), OCT4 (Abcam, ab18976, 1:200) and CDX2 (BioGenex, AM392, 1:50) at room temperature for 1 h. After washed 3 times in 45 min with 0.3% PVP in DPBS, embryos were incubated for 1 h with secondary antibodies including goat anti-Rabbit IgG conjugated with Alexa Fluor 488 (Molecular probe, A11008, 1:200) or goat anti-mouse IgG conjugated with Alexa Fluor 488 (Molecular probe, A11029, 1:200) in the dark at RT. Finally, embryos were washed several times and mounted on glass slides with a small drop of Vectashield (VectorLab) mounting medium, then covered by a glass coverslip and imaged using confocal laser scanning microscopy (Olympus, FluoView1000). Embryos stained without primary antibody were used as negative controls to examine the specificity of the reaction.

### Quantitative analysis of fluorescence intensity

The signal intensity for 5mC and H3K9me2 in embryos was analyzed as described previously[17]. Briefly, nuclei of blastomeres were identified by DAPI staining using the 40 × objective lens. Using the same setting involved magnification fold, objective, exposure, brightness and contrast, fluorescence images were captured by Olympus FluoView 1000 software. Quantitative assessment of signal intensity in nuclei or cytoplasmic areas was performed using Image J software. The border around the nuclei was manually delineated according to DNA staining. In addition, at least three different cytoplasmic areas were delineated for normalization to background. The average pixel intensity of the nuclear areas was calculated by Image J, and then normalized by dividing by the average pixel intensity of the background areas.

### Statistical analysis

All experiments were repeated at least three times. Except special instructions, the data were presented as mean ± standard error of mean (mean ± S.E.M) and SPSS (Version 17.0) was used to do single factor analysis of variance (ANOVA) of the cleavage rate, blastocyst rate, total cell number per blastocyst, fluorescence intensity of 5mC and H3K9me2. *P<*0.05 was considered statistically significant.

## Results

### TSA treatment alone improves early developmental efficiency of SCNT embryos

**Fig 1:**
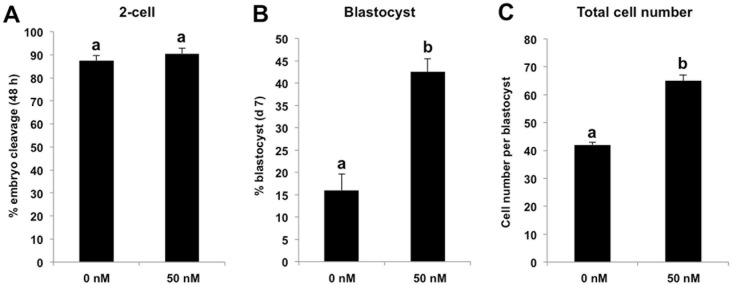
TSA treatment alone improves early developmental efficiency of SCNT embryos. SCNT embryos at the pronuclear stage were cultured in PZM-3 supplemented with 50 nM TSA for 24 h and embryos cultured in PZM-3 containing DMSO were served as NT control, then TSA and DMSO were washed out and embryos were cultured in fresh PZM-3 medium until day 7. The effect of HDACs inhibition on the proportion of embryos that reached the 2-cell stage within 48 h after activation **(A)**, proportion of embryos developing to the blastocyst stage (Day 7) **(B)** and total cell number per blastocyst **(C)** are shown. The experiment was repeated at least three times with 200 embryos per group. All the percentage data are expressed relative to the number of 1-cell embryos and are shown as mean ± S.E.M. Values with different superscripts across treatments indicate significant differences (*P* < 0.05).

Our previous study has shown that TSA improved the early developmental rate of porcine SCNT embryos under a specific concentration and duration condition [10]. Therefore, we cultured SCNT embryos in PZM-3 medium supplemented with 50 nM TSA for 24 h, and then TSA were washed out and embryos were cultured in fresh PZM-3 medium until day 7. We found that TSA treatment did not change the cleavage rate of SCNT embryos (Fig 1A), but it significantly enhanced the blastocyst rate and total cell number per blastocyst compared with control group (*P* < 0.05) (Fig 1B and 1C). Hence, these data suggest that TSA treatment is sufficient to elevate the early developmental efficiency of porcine SCNT embryos.

### BIX-01294 treatment alone enhances early developmental efficiency of SCNT embryos

**Fig 2:**
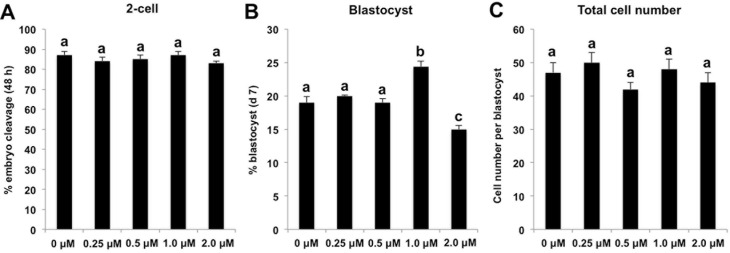
BIX-01294 treatment alone enhances early developmental efficiency of SCNT embryos. SCNT embryos at the pronuclear stage were cultured in PZM-3 supplemented with different concentrations of BIX-01294 at 0 (embryo culture water as control), 0.25, 0.5, 1.0 and 2.0 μM for 24 h, then BIX-01294 in different treatment groups were washed out and embryos were cultured in fresh PZM-3 medium until day 7. The effect of G9A inhibition on the proportion of embryos that reached the 2-cell stage within 48 h after activation **(A)**, proportion of embryos developing to the blastocyst stage (Day 7) **(B)** and total cell number per blastocyst **(C)** are shown. The experiment was repeated at least three times with 130 embryos per group. All the percentage data are expressed relative to the number of 1-cell embryos and are shown as mean ± S.E.M. Values with different superscripts across treatments indicate significant differences (*P* < 0.05).

Two recent studies demonstrated that knockdown or BIX-01294-mediated inactivation of histone methyltransferas G9a that catalyzes histone H3K9 dimethylation could significantly improve the cloning efficiency in mouse and pig[20,21]. To investigate whether combined treatment of TSA and BIX-01294 synergistically improved the early developmental competence of porcine cloned embryos, we first examined the effect of BIX-01294 treatment alone on the early developmental efficiency of cloned embryos under our experimental conditions. We first cultured SCNT embryos in PZM-3 medium supplemented with different concentrations of BIX-01294 at 0 (embryo culture water as control), 0.25, 0.5, 1.0 and 2.0 μM for 24 h, then BIX-01294 was washed out and embryos were cultured in fresh PZM-3 medium until day7. We found that there was no difference in the cleavage rate and total cell number per blastocyst among various groups (Fig 2A and 2C), but the blastocyst rate in 1.0 μM BIX-01294 group was significantly higher than other groups (*P* < 0.05) (Fig 2B). Therefore, these data indicate that BIX-01294 improves the early developmental efficiency of porcine SCNT embryos under a specific concentration condition.

### TSA and BIX-01294 treatment combined enhances early developmental efficiency of SCNT embryos

**Fig 3:**
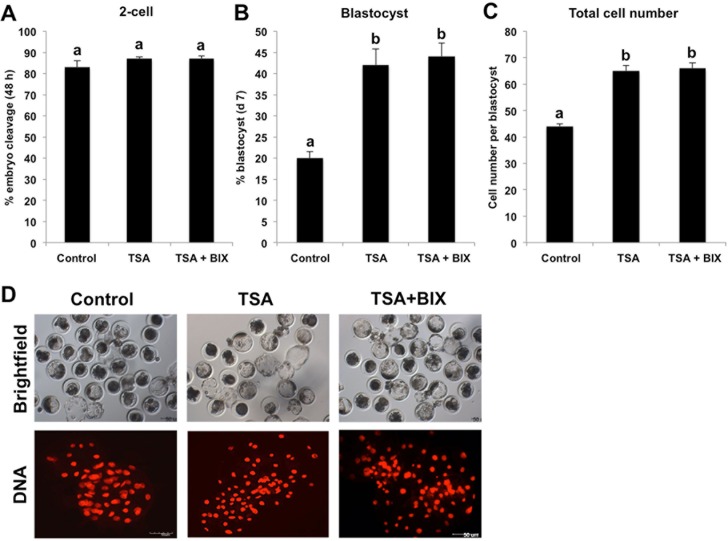
TSA and BIX-01294 treatment combined enhances early developmental efficiency of SCNT embryos. SCNT embryos at the pronuclear stage were cultured in PZM-3 supplemented with 50 nM TSA or 50 nM TSA and 1 μM BIX-01294 for 24 h and embryos cultured in PZM-3 containing DMSO were served as NT control, then DMSO, TSA and BIX-01294 in different groups were washed out and embryos were cultured in fresh PZM-3 medium until day7. The effect of HDACs and G9A inhibition on the proportion of embryos that reached the 2-cell stage within 48 h after activation **(A)**, proportion of embryos developing to the blastocyst stage (Day 7) **(B)** and total cell number per blastocyst **(C)** are shown. The experiment was repeated at least three times with 220 embryos per group. All the percentage data are expressed relative to the number of 1-cell embryos and are shown as mean ± S.E.M. Values with different superscripts across treatments indicate significant differences (*P* < 0.05). **(D)** Representative images of SCNT blastocysts from different groups under bright field. DNA was labeled with propidium iodide (red). Scale bars = 50μm.

Given the mutual crosstalk of histone deacetylation and methylation can further reinforce chromatin compaction that may repress expression of genes related with early embryonic development, we speculated that simultaneous inhibition of HDACs and histone methyltranseferase could synergistically promote early developmental rate of porcine SCNT embryos. To test this hypothesis, we cultured SCNT embryos in PZM-3 medium supplemented with 50 nM TSA and 1 μM BIX-01294 for 24 h, whereas embryos cultured within PZM-3 containing 50 nM TSA as NT positive control and embryos cultured within PZM-3 without any supplements served as NT negative control. After 24 h incubation, embryos from three groups were cultured in fresh PZM-3 medium until day 7. We found that the cleavage rate is similar among three groups (Fig 3A), but combined treatment with TSA and BIX-01294 or TSA treatment alone could significantly elevate the blastocyst rate and total cell number per blastocyst compared with NT negative control (*P* < 0.05) (Fig 3B, 3C and 3D). Unfortunately, combined treatment with TSA and BIX-01294 did not further elevate the blastocyst rate and quality compared with TSA treatment alone. Collectively, these data suggest that combined treatment with TSA and BIX-01294 can enhance early developmental efficiency of porcine SCNT embryos.

### TSA and BIX-01294 treatment combined corrects abnormal H3K9me2 and 5mC levels in SCNT embryos

**Fig 4:**
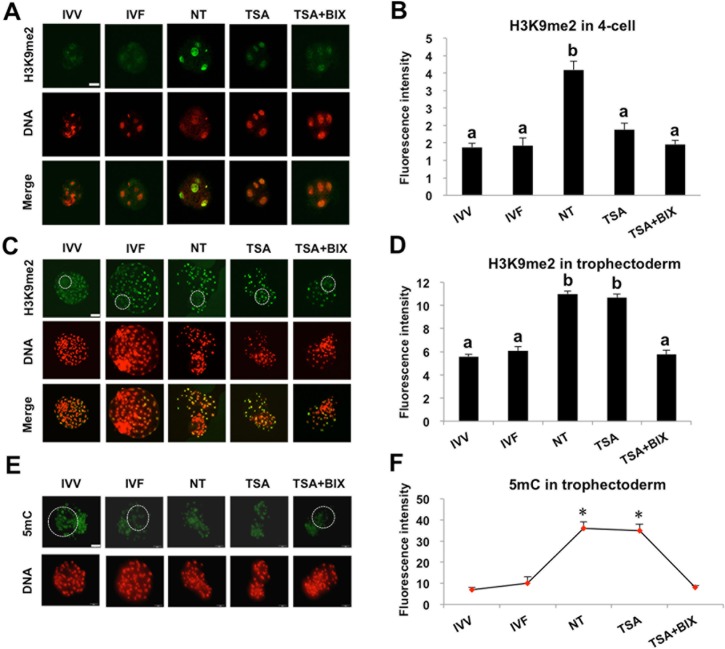
TSA and BIX-01294 treatment combined restores H3K9me2 and 5mC levels in SCNT embryos. SCNT embryos at the pronuclear stage were cultured in PZM-3 supplemented with 50 nM TSA or 50 nM TSA and 1 μM BIX-01294 for 24 h and SCNT embryos cultured in PZM-3 containing DMSO were served as NT control. Then DMSO, TSA and BIX-01294 in different groups were washed out and embryos were cultured in fresh PZM-3 medium until day7. We isolated and fixed the 4-cell and blastocysts from different groups including NT control, TSA treatment, TSA and BIX-01294 combination treatment for subsequent experiments. At the same time, *in vivo* fertilized (IVV) and *in vitro* fertilized (IVF) 4-cell and blastocysts were also collected and fixed. Finally, 4-cell embryos **(A)** and blastocysts **(C)** were stained for H3K9me2 (green) and DNA (propidium iodide, red). Bottom panels showed the merged images (yellow) between H3K9me2 signals (green) and DNA staining (red). **(E)** Blastocysts from all groups were stained for 5mC (green) and DNA (propidium iodide, red). Shown in A, C and E are representative Z-sections obtained by confocal microscopy from one experiment. White dash circle denotes ICM area in **C** and TE region in **E**, respectively. Scale bars = 50μm. **(B, D)** Quantification of the H3K9me2 fluorescence intensity in nucleus of 4-cell and trophectoderm cells. **(F)** Quantification of the 5mC fluorescence intensity in nucleus of trophectoderm cells. Data in B, D and F are shown as mean ± S.E.M. All experiments above were repeated three times with 20 embryos per group. Values with different superscripts across groups indicate significant differences (*P* < 0.05) and *P < 0.05.

Because we previously observed the abnormally high levels of H3K9me2 and 5mC in 4-cell and trophectoderm (TE) lineage of somatic cloned blastocysts as compared with IVF counterparts in our published study[17], we attempted to explore whether combined treatment with TSA and BIX-01294 could correct the expression levels of these two epigenetic markers in TSA and/or BIX-01294 treated-SCNT embryos. To address this question, H3K9me2 and 5mC levels were evaluated by immunofluorescence and quantitative analysis in embryos derived from *in vivo* fertilized (IVV), IVF, non-treatment (NT control), and TSA treatment alone and TSA and BIX-01294 treatment combined. IVV and IVF embryos were used as positive control. In line with a previous report [17], H3K9me2 level in 4-cell and TE lineage of SCNT blastocysts was significantly higher than that in IVV and IVF counterparts (*P* < 0.05) (Fig 4A, 4B, 4C and 4D). By contrast, H3K9me2 level was significantly reduced in 4-cell and TE lineage of SCNT blastocysts in combined treatment of TSA and BIX-01294, which was comparable with that in IVV and IVF embryos (Fig 4A, 4B, 4C and 4D). Surprisingly, TSA treatment alone only rescued the expression level of H3K9me2 in 4-cell but did not correct the aberrant expression level of H3K9me2 in TE lineage of blastocysts (Fig 4C and 4D). Analogously, 5mC level in TE lineage of blastocysts from NT control and TSA treatment alone was apparently higher than that in IVV and IVF counterparts (*P* < 0.05) (Fig 4E and 4F). Conversely, combined treatment of TSA and BIX-01294 could significantly reduce the expression level of 5mC in TE lineage of NT blastocysts, which was similar to that in IVV and IVF counterparts (Fig 4E and 4F). Altogether, these results demonstrate that combined treatment with TSA and BIX-01294 restores H3K9me2 and 5mC levels of somatic cloned embryos to natural reprogramming state.

### TSA and BIX-01294 treatment combined increases abundance of proteins associated with lineage differentiation in SCNT blastocysts

**Fig 5:**
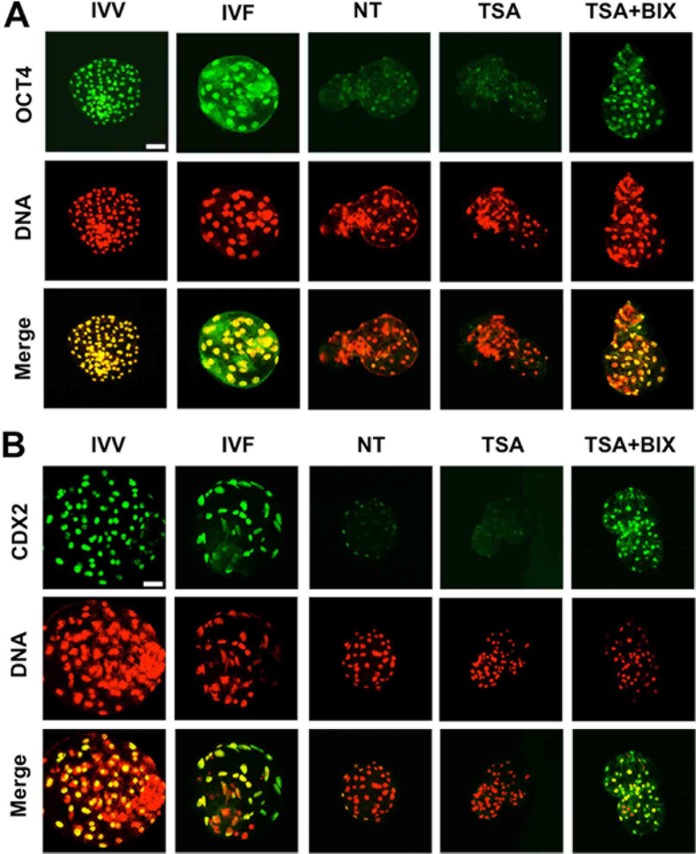
TSA and BIX-01294 treatment combined increases expression of proteins associated with lineage differentiation in SCNT blastocysts. SCNT embryos at the pronuclear stage were cultured in PZM-3 supplemented with 50 nM TSA or 50 nM TSA and 1 μM BIX-01294 for 24 h and SCNT embryos cultured in PZM-3 containing DMSO were served as NT control. Then DMSO, TSA and BIX-01294 in different groups were washed out and embryos were cultured in fresh PZM-3 medium until day 7. We collected and fixed blastocysts from different groups including NT control, TSA treatment, TSA and BIX-01294 combination treatment for subsequent experiments. At the same time, *in vivo* fertilized (IVV) and *in vitro* fertilized (IVF) blastocysts were also collected and fixed. Finally, blastocysts were stained for OCT4 **(A)** or CDX2 **(B)** (green) and DNA (propidium iodide, red). Bottom panels showed the merged images (yellow) between OCT4/CDX2 signals (green) and DNA staining (red). Two experiments above were repeated three times with 20 embryos per group. Scale bars = 50μm.

To further investigate the molecular mechanisms underlying the increase in the developmental capacity of somatic cloned embryos after combined treatment of TSA and BIX-01294, we evaluated the expression of OCT4 and CDX2 protein related with the differentiation of inner cell mass (ICM) and trophectoderm (TE) lineage through immunofluorescence and quantitative analysis at the blastocyst stage. IVV and IVF embryos were used as positive control. As expected, the expression levels of OCT4 and CDX2 were significantly increased after only combined treatment of TSA and BIX-01294, reaching the comparable levels observed in IVV and IVF counterparts (Fig 5A and 5B). However, TSA treatment alone did not exert the beneficial effects (Fig 5A and 5B). Therefore, these data indicate that combined treatment of TSA and BIX-01294 facilitates the increased expression of proteins relevant to differentiation of ICM and TE lineage in SCNT blastocysts.

## Discussion

**Fig 6:**
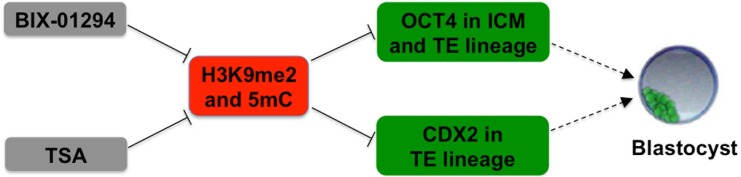
Model for the potential mechanisms of TSA and BIX-01294-induced the enhanced developmental efficiency of SCNT embryos. Histone deacetylases and methyltransferase inhibitor (TSA and BIX-01294) synergistically reduce the expression levels of H3K9me2 and 5mC in SCNT 4-cell and blastocysts which could indirectly promote the increased expression of OCT4 and CDX2 important for both inner cell mass (ICM) and trophectoderm (TE) lineage differentiation. Based on these data, we propose that TSA and BIX-01294 enhance blastocyst formation rate of porcine SCNT embryos potentially through facilitating the increased expression of OCT4 and CDX2 involved in the differentiation of ICM and TE lineage. Solid and dash lines indicate the proven and putative results in the present study, respectively.

A number of previous studies showed that SCNT manipulation often incurs aberrant DNA and histone modifications that could contribute to the poor cloning efficiency [6,12,19,24]. Therefore, optimization in epigenetic states of SCNT embryos through exposure to chemical drugs has been used as a common strategy in improvements of cloning efficiency. For example, histone hyperacetylation induced by HDACs inhibitor TSA could significantly improve the full-term developmental rate in pig[25]. Decreasing the H3K9me2 level induced by BIX-01294 treatment also could elevate the porcine cloning efficiency [21]. However, the early and full-term developmental efficiency of SCNT embryos is still low compared with naturally fertilized embryos. Recently, we characterized the dynamic expression patterns of DNA methylation (5mC) and H3K9me2 during early development of porcine SCNT and IVF embryos. The results showed that somatic cloned embryos have higher levels of DNA methylation and H3K9me2 in 4-cell and trophectoderm lineage of blastocysts compared with those derived from IVF [17,22]. Based on these data, we hypothesized that TSA and BIX-01294 treatment can correct the abnormal DNA and histone methylation to improve the early developmental efficiency of porcine cloned embryos. In the present study we found that TSA and/or BIX-01294 treatment, alone and in combination, significantly improved the early developmental competence of cloned embryos. Furthermore, combined treatment of TSA and BIX-01294 not only reduced the 5mC and H2K9me2 levels in 4-cell and trophectoderm lineage of blastocysts, but also increased the expression levels of OCT4 and CDX2. Thus, we propose a model in which combined treatment of TSA and BIX-01294 improves the blastocyst fate and quality via reducing the 5mC and H3K9me2 levels and increasing the expression levels of OCT4 and CDX2 relevant to the differentiation of ICM and TE lineage (Fig 6).

Trichostatin A (TSA) is an inhibitor of histone deacetylase that potentially increase the global acetylation level in cloned embryos. Based on our previous study, 50 nM TSA was used to treat SCNT embryos due to this concentration of TSA did not impair the early development of porcine somatic cloned embryos [10]. The results showed that TSA treatment improved *in vitro* developmental efficiency and quality of porcine SCNT embryos, which is consistent with several previous studies [11,25]. However, the dosage of TSA used in the study is different from that in other studies (50 nM vs.37.5 nM or 5 nM) [11,26]. This may be due to these differences in types of donor cells, nuclear transfer methods and period of TSA exposure. Anyway, the concentration and exposure duration of TSA we used should be appropriate as long as it does not alter the normal cell cycle progression and cellular activity. On the other hand, BIX-01294 has been proved to be a potent inhibitor of G9A that reduces H3K9me2 level in somatic cells [27]. In the present study, we observed that 1 μM BIX-01294 treatment alone promoted the rate of blastocyst formation of porcine cloned embryos. However, a recent study reported that 5 nM and 50 nM BIX-01294 treatment significantly improved *in vitro* development of porcine somatic cloned embryos[21]. These specific reasons underlying the discrepancy need to be further explored in future. Treatment of lower concentration (0.25 μM and 0.5 μM) does not affect the developmental rate and quality, whereas BIX-01294 exposure with higher concentration (2 μM) impaired the developmental competence of cloned embryos. These findings are largely consistent with those observed in pig [10] and sheep [28], which showed that higher concentration caused the reduced developmental capacity and even embryonic lethality. This detrimental effect of BIX-01294 treatment at higher concentration might be attribute to the complete loss of G9A activity, as G9a-null mice exhibits embryonic lethality shortly after implantation[29].

Due to the observed beneficial effects of TSA or BIX-01294 treatment alone in improving the early developmental rate of porcine somatic cloned embryos, we asked whether combined treatment of TSA and BIX-01292 synergistically promotes the early development of SCNT embryos. The results of the present study showed that there was not different in the rate of blastocyst formation and total cell number per blastocyst between TSA treatment alone and two inhibitors treatment in combination, suggesting that combined treatment did not further improve the early developmental competence of porcine cloned embryos. A recent study showed that BIX-01294 treatment reduced the overall histone acetylation but another HDACs inhibitor Scriptaid restored the histone acetylation states in porcine cloned embryos [21]. In addition, previous studies also demonstrated that TSA treatment could indeed significantly increase the global histone acetylation levels in porcine somatic cloned embryos[11]. Hence, the reason for invisible synergistic effects for early embryonic development after combined treatment of TSA and BIX-01294 might be due to that TSA-induced higher histone acetylation was used to compensate for the reduced acetylation level after BIX-01294 exposure.

Interestingly, although global histone acetylation levels in combined treatment group is supposed to be lower than that in TSA treatment alone group, developmental efficiency and blastocyst quality is comparable between combined treatment and TSA treatment alone. This prompted us to examine whether abnormal 5mC and histone H3K9me2 observed in cloned embryos was restored to the normal state after combined treatment. Accordingly, these analysis revealed that H3K9me2 level at SCNT 4-cell stage after combined treatment was reduced to the normal state at *in vivo* and *in vitro* fertilized 4-cell stage corresponding to porcine embryo genomic activation, which is consistent with that observed in other studies [21,30]. Unexpectedly, TSA treatment alone could also reduce the H3K9me2 level to that in naturally fertilized 4-cell, which might be due to HDACs inhibitor induced the decreased H3K9me2 level[31]. However, only combined treatment could rescue the expression levels of 5mC and H3K9me2 in TE lineage of blastocysts, but TSA treatment alone did not exert this effect at blastocyst stage, which suggests that only BIX-01294 exposure can correct the 5mC and H3K9me2 states in porcine SCNT blastocysts. DNA and histone demethylation /remethyaltion is involved in epigenetic reprogramming [32] and transcriptional regulation of genes important for lineage specification and differentiation in early embryonic development [33]. Because G9a-mediated H3K9me2 provide a docking site for binding protein HP1 that dramatically recruits DNA methyltransferase DNMT1 to methylate the downstream genes [34]. In the present study, BIX-01294 could potentially utilize this mechanism to reduce the global 5mC level in TE lineage of cloned blastocysts.

Epigenetic changes have to be translated into functionally transcriptional expression that mainly involved in regulation of early embryogenesis. To further explore the effect of combined treatment-mediated the reduced 5mC and H3K9me2 on SCNT embryo development, we examined protein expression of two key transcription factors including OCT4 and CDX2 at the blastocyst stage after combined treatment or TSA treatment alone. The reduced expression of OCT4 and CDX2 in porcine somatic cloned blastocysts compared with IVF counterparts has been reported in the previous studies [26,35]. Analogous to this, we observed that expression levels of OCT4 and CDX2 in SCNT blastocysts were lower than that in *in vivo* and *in vitro* fertilized counterparts. In contrast, combined treatment of TSA and BIX-01294 significantly improved the expression of these two proteins in ICM and TE lineages of SCNT blastocysts. OCT4 is required for the specification of inner cell mass lineage in mouse [36] as well as blastocyst formation in pig [37]. Furthermore, DNA and H3K9 demethylation could enhance the reprogramming efficiency and pluripotency through inducing the increased expression of *OCT4* [21,38]. In mouse preimplantation embryos Cdx2 is essential for the specification of TE lineage and blastocyst formation and expansion [39]. Therefore, the increased expression of OCT4 and CDX2 could contribute to the improvements in developmental efficiency of porcine SCNT embryos.

Taken together, these data reported here indicated that combined treatment of TSA and BIX-01294 improved early developmental competence of porcine SCNT embryos potentially via improvements in epigenetic status and protein expression.
